# Wnt signaling blockage inhibits cell proliferation and migration, and induces apoptosis in triple-negative breast cancer cells

**DOI:** 10.1186/1479-5876-11-280

**Published:** 2013-11-04

**Authors:** Birdal Bilir, Omer Kucuk, Carlos S Moreno

**Affiliations:** 1 Department of Pathology and Laboratory Medicine, Emory University School of Medicine, Atlanta, Georgia, USA; 2 Department of Hematology and Medical Oncology, Emory University School of Medicine, Atlanta, Georgia, USA; 3 Winship Cancer Institute, Emory University, Atlanta, Georgia, USA

**Keywords:** Triple-negative breast cancer, Wnt signaling pathway, iCRT-3, SOX4

## Abstract

**Background:**

Triple-negative breast cancer (TNBC) is an aggressive clinical subtype of breast cancer that is characterized by the lack of estrogen receptor (ER) and progesterone receptor (PR) expression as well as human epidermal growth factor receptor 2 (HER2) overexpression. The TNBC subtype constitutes approximately 10%–20% of all breast cancers, but has no effective molecular targeted therapies. Previous meta-analysis of gene expression profiles of 587 TNBC cases from 21 studies demonstrated high expression of Wnt signaling pathway-associated genes in basal-like 2 and mesenchymal subtypes of TNBC. In this study, we investigated the potential of Wnt pathway inhibitors in effective treatment of TNBC.

**Methods:**

Activation of Wnt pathway was assessed in four TNBC cell lines (BT-549, MDA-MB-231, HCC-1143 and HCC-1937), and the ER^+^ cell line MCF-7 using confocal microscopy and Western blot analysis of pathway components. Effectiveness of five different Wnt pathway inhibitors (iCRT-3, iCRT-5, iCRT-14, IWP-4 and XAV-939) on cell proliferation and apoptosis were tested *in vitro*. The inhibitory effects of iCRT-3 on canonical Wnt signaling in TNBC was evaluated by quantitative real-time RT-PCR analysis of Axin2 and dual-luciferase reporter assays. The effects of shRNA knockdown of SOX4 in combination with iCRT-3 and/or genistein treatments on cell proliferation, migration and invasion on BT-549 cells were also evaluated.

**Results:**

Immunofluorescence staining of β-catenin in TNBC cell lines showed both nuclear and cytoplasmic localization, indicating activation of Wnt pathway in TNBC cells. iCRT-3 was the most effective compound for inhibiting proliferation and antagonizing Wnt signaling in TNBC cells. In addition, treatment with iCRT-3 resulted in increased apoptosis *in vitro*. Knockdown of the Wnt pathway transcription factor, SOX4 in triple negative BT-549 cells resulted in decreased cell proliferation and migration, and combination treatment of iCRT-3 with SOX4 knockdown had a synergistic effect on inhibition of cell proliferation and induction of apoptosis.

**Conclusions:**

These data suggest that targeting SOX4 and/or the Wnt pathway could have therapeutic benefit for TNBC patients.

## Background

Breast cancer is the most common malignancy and the second leading cause of cancer death among women in the United States. About 226,870 new cases of breast cancer and 39,510 breast cancer deaths are estimated to occur among US women in 2012 [1]. It is a heterogeneous disease comprised of distinct subtypes based on clinical, pathological, and genetic findings [2]. One of these subtypes is triple-negative breast cancer (TNBC), which is characterized by lack of expression of estrogen receptor (ER) and progesterone receptor (PR) as well as absence of human epidermal growth factor receptor 2 (HER2) overexpression [3]. TNBC accounts for approximately 10%-20% of all breast cancer cases [3-5]. TNBC patients exhibit a more aggressive clinical course, and have a higher rate of distant recurrence and a poorer prognosis than women with other breast cancer subtypes [3,6,7]. Population-based studies identified several risk factors for TNBC including young age at diagnosis, African-American race, high body mass index, young age at menarche, high parity, young age at time of first birth, and lack of breast feeding [3-5]. TNBC tumors are typically larger in size and of higher grade [8]. Due to the lack of well-defined molecular targets in TNBC, chemotherapy remains the mainstay of systemic treatment for this disease [9].

Cluster analysis of gene expression profiles from 587 TNBC cases identified six distinct subtypes, including two basal-like, an immunomodulatory, a mesenchymal, a mesenchymal stem-like, and a luminal androgen receptor subtype [10]. Signaling pathway abnormalities implicated in the pathogenesis of TNBC include DNA damage response, apoptosis, proliferation, epithelial-mesenchymal transition, immune response, and angiogenesis [10,11].

Several components of the Wnt pathway as well as the Myc pathway are upregulated in the basal-like 2 and mesenchymal subtypes of TNBC such as WNT5A, SOX11, SOX4, LRP6, FZD4, and FZD7 [10,12,13]. Wnt signaling regulates cell proliferation, survival, and differentiation, and plays key roles in embryonic development and tumorigenesis [14-17]. In the absence of Wnt ligands, cytoplasmic β-catenin is recruited into a destruction complex that consists of adenomatous polyposis coli (APC), glycogen synthase kinase-3β (GSK3β), axin, and casein kinase 1 (CK1). This complex formation induces the phosphorylation of β-catenin at the amino terminus by GSK3β and CK1, resulting in the ubiquitination and the subsequent degradation of β-catenin [16-19]. When Wnt ligands are secreted, they bind to their receptors, low-density lipoprotein receptor-related protein 5/6 (LRP5/6) and Frizzled (FZD), to activate the Wnt signaling pathway. This binding leads to the recruitment of the scaffolding protein Dishevelled (Dsh) and axin to the cell membrane, and inactivation of the destruction complex. Inhibition of the degradation of β-catenin allows the cytoplasmic stabilization and translocation of the protein to the nucleus where it binds to members of the T-cell factor/lymphoid enhancing factor (TCF/LEF) family of transcription factors, and induces the expression of Wnt target genes. Sex-determining region Y-box 4 (SOX4), which is a highly conserved developmental transcription factor, has been implicated in playing an important role in Wnt signaling pathway in cancers [20,21]. SOX4 contains a high-mobility group DNA-binding domain that is structurally related to TCF/LEFs [22]. Sinner *et al*. demonstrated that SOX4 stabilizes β-catenin, and enhances Wnt signaling pathway in colon carcinoma [23], while Scharer *et al*. demonstrated cooperativity between SOX4 and β-catenin in prostate cancer cells [21]. SOX4 was also shown to have a role in Wnt signaling in malignant melanoma by regulating β-catenin [24,25].

Genistein, the major isoflavone in soybean, has been shown to have anticancer effects [26-28]. Genistein treatment of prostate cancer cells upregulates the expression of GSK3β, enhances GSK3β binding to β-catenin, and increases β-catenin phosphorylation, resulting in inactivation of Wnt/β-catenin and inhibiting cancer growth [29,30]. Genistein was also shown to diminish Wnt1-induced proliferation and decrease the expression of Wnt targets, namely c-myc and cyclin D1 [31,32]. Genistein also suppresses β-catenin transcriptional activity in colorectal carcinoma cells, and reduces cell proliferation through theWnt pathway in mesenchymal stromal cells isolated from human umbilical cord [33,34].

Other recently identified inhibitors of the Wnt pathway include the inhibitors of catenin-responsive transcription (iCRT) compounds, iCRT-3, iCRT-5, and iCRT-14, which were identified in an RNAi modifier screen [35]. The Novartis compound XAV-939 stimulates β-catenin degradation by inhibiting Tankyrase and thereby stabilizing axin [36]. The IWP-4 compound was identified in a small molecule screen for Wnt antagonists and inhibits Porcupine, the membrane-bound acyltransferase that is essential to the pamitoylation of Wnt ligands [37]. In this study, we investigated the potential of these Wnt pathway inhibitors in effective treatment of TNBC cells. In addition, we tested the effects of reduced SOX4 levels in combination with Wnt inhibitor and/or the soy isoflavone genistein treatment on cell proliferation, migration and invasion on TNBC cells. We found that iCRT-3 and SOX4 knockdown have potential for therapy of TNBC.

## Methods

### Cell culture and reagents

MCF-7 and TNBC cell lines (BT-549, MDA-MB-231, HCC-1143 and HCC-1937) were obtained from American Type Culture Collection (Manassas, VA). MCF-7 and MDA-MB-231 cells were maintained in DMEM (Cellgro, Manassas, VA) while BT-549, HCC-1143 and HCC-1937 cells were grown in RPMI 1640 medium (Gibco, Grand Island, NY). Both media were supplemented with 10% fetal bovine serum (FBS) (Atlanta Biologicals, Lawrenceville, GA), 2 mM L-Glutamine (Gibco, Grand Island, NY) and 50 U/ml Penicillin-50 μg/ml Streptomycin antibiotics (Gibco, Grand Island, NY). Medium for BT-549 was also supplemented with 0.023 IU/ml bovine insulin (Sigma-Aldrich, St. Louis, MO). Cell lines were cultured in a 37°C incubator with humidified atmosphere of 5% CO_2_.

XAV-939 and genistein were purchased from Sigma-Aldrich (St. Louis, MO). iCRT-3, iCRT-5 and iCRT-14 were obtained from ChemDiv (San Diego, CA). IWP-4 was purchased from Stemgent (San Diego, CA). Each compound was reconstituted in dimethyl sulfoxide (DMSO) (EMD, Germany). Recombinant human Wnt-3a (5036-WNP) was purchased from R&D Systems (Minneapolis, MN), and reconstituted in PBS containing 0.1% BSA. Puromycin was obtained from Enzo Life Sciences (Farmingdale, NY). Matrigel was purchased from BD Biosciences (San Jose, CA). Trypan blue solution was obtained from Thermo Scientific (Waltham, MA). Rabbit anti-SOX4 (LS-B3520) antibody, mouse monoclonal antibody against active β-catenin (clone 8E7, #05-665), and mouse monoclonal antibody against β-actin (8H10D10, #3700) were purchased from LifeSpan BioSciences (Seattle, WA), Millipore (Billerica, MA) and Cell Signaling Technology (Danvers, MA), respectively. Mouse monoclonal antibody against β-catenin (E-5, sc-7963) and rabbit polyclonal antibody against Dvl-2 (H-75, sc-13974) were procured from Santa Cruz Biotechnology, Inc (Santa Cruz, CA). IRDye 680RD goat anti-rabbit and IRDye 800CW goat anti-mouse secondary antibodies were purchased from LI-COR Biosciences (Lincoln, NE). Alexa Fluor® 488 conjugated goat anti-mouse secondary antibody (A11017) and Hoechst 33342 (H3570) were procured from Molecular Probes (Eugene, OR). Fluoromount-G medium was purchased from SouthernBiotech (Birmingham, AL).

### Immunofluorescence staining and confocal microscopy

Cells were grown on sterile coverslips placed in 6-well plate, and serum-starved for 24 hours prior to the treatment with 200 ng/ml Wnt-3a for 4 hours. Cells were then fixed with 4% paraformaldehyde for 15 minutes at room temperature, and permeabilized with 0.5% Triton X-100 for 10 minutes. After blocking with 3% BSA for 30 minutes, cells were incubated with the primary antibody (mouse monoclonal antibody against β-catenin, 1:100 dilution) for overnight at 4°C. Cells were then incubated with Alexa Fluor® 488 conjugated goat anti-mouse secondary antibody at 1:1,000 dilution for 1 hr at room temperature in the dark. To ensure specificity of our results, negative controls with no primary antibody or no secondary antibody were included. For nuclear counterstaining, cells were incubated with Hoechst 33342 (1:10,000 dilution) for 15 minutes. Coverslips were then mounted with Fluoromount-G. Cells were visualized using Zeiss LSM510 Meta confocal microscope (Carl Zeiss Microscopy GmbH, Germany). Images were acquired at 200× total magnification using Zeiss Zen 2009 software.

### Generation of stable SOX4 knockdown cell line

Knockdown of SOX4 expression was performed using MISSION short hairpin RNA (shRNA) lentiviral transduction particles (Sigma-Aldrich, St. Louis, MO) according to manufacturer’s protocol. BT-549 cells were transduced with scrambled control shRNA or SOX4 shRNA lentiviral construct at 60-70% confluency. Puromycin (1 μg/ml) was administered for two weeks for selection of transduced cells. Knockdown of SOX4 was verified by Western blotting and quantitative real-time RT-PCR.

### Western blot analysis

Whole cell lysates were prepared from cells on 100 mm-culture dish in lysis buffer containing 137 mM NaCl, 20 mM Tris–HCl (pH 8.0), 10% glycerol, 1% NP-40, and protease inhibitor cocktail (Promega, San Luis Obispo, CA). Protein concentrations in the supernatants were determined using Micro BCA Protein Assay Kit (Pierce Biotechnology, Rockford, IL). 50 μg total protein was separated on 7.5 or 10% SDS-polyacrylamide gel, and transferred to nitrocellulose membrane. Membrane was blocked in 1× PBS buffer containing 0.1% Tween-20 and 5% non-fat dry milk for 1 hour at room temperature, and then incubated with primary antibody (mouse anti-active β-catenin, 1:1,000; rabbit anti-Dvl-2, 1:400; rabbit anti-SOX4, 1:1,000) overnight at 4°C. Mouse monoclonal antibody against β-actin (1:5,000) was used as normalization control. Membrane was then incubated with fluorescence-conjugated secondary antibodies (IRDye 680RD goat anti-rabbit and IRDye 800CW goat anti-mouse) at 1:5,000 dilution for 1 hour at room temperature, and signals were visualized and quantitated using the Odyssey infrared imaging system (LI-COR Biosciences, Lincoln, NE). Immunoblots were repeated three times with new lysates from independent experiments.

### Quantitative real-time RT-PCR analysis

Total RNA was extracted from cultured cells using the RNeasy Mini Kit (Qiagen, Valencia, CA), quantitated using NanoDrop 1000 (NanoDrop, Wilmington, DE), and reverse transcribed into cDNA using iScript cDNA Synthesis Kit (Bio-Rad Laboratories, Hercules, CA). Quantitative real-time PCR was performed using iQ SYBR Green Supermix (Bio-Rad Laboratories) on a Bio-Rad iCycler. Sequences of the primers for Axin2 were 5′-CAGGACACTGCTCTCTCAGATTCA-3′ (forward) and 5′-TCACAACAGCCTTTGCAGGG-3′ (reverse). Sequences of the primers for SOX4 were 5′-CCGAGCTGGTGCAAGACC-3′ (forward) and 5′-CCACACCATGAAGGCGTTC-3′ (reverse). Sequences of the primers for β-actin were 5′-CTGGAACGGTGAAGGTGACA-3′ (forward) and 5′-AAGGGACTTCCTGTAACAATGCA-3′ (reverse). The relative changes in gene expression data were analyzed by the 2^-ΔΔCT^ method. β-actin was used as an internal control. Triplicates were run for each sample in three independent experiments.

### Apoptosis assay

Analysis of apoptosis was performed using Caspase-Glo 3/7 assay (Promega) according to manufacturer’s protocol. BT-549 cells that were transduced with scrambled or SOX4 shRNA lentiviral particles were seeded in 96-well plate, and incubated overnight. Cells were then treated with DMSO or 25 μM iCRT-3 for 12 hours. Caspase 3/7 activity was measured using FLUOstar OPTIMA (BMG Labtech, Cary, NC) microplate reader. Each sample was assayed in triplicate in three independent experiments.

### Dual-luciferase reporter assay

BT-549 cells were seeded into 12-well plates. After overnight incubation, cells were transiently transfected with 0.5 μg of TOP-FLASH firefly luciferase reporter vector (Promega) and 0.04 μg of *Renilla* luciferase vector (Promega) as an internal control for transfection efficiency using Lipofectamine 2000 (Invitrogen) according to the manufacturer’s protocol. After 24 hour-transfection, cells were treated with DMSO or 25 μM iCRT-3 for 48 hours. Cells were then lysed, and luciferase activities were measured using Dual-Luciferase Reporter Assay System (Promega) and TD-20/20 luminometer (Turner Design). The relative luciferase activity was calculated by firefly luciferase activity/*Renilla* luciferase activity. Data were presented as mean ± SEM from three independent experiments.

### Cell proliferation, migration, and invasion assays using xCELLigence system

xCELLigence experiments were performed using the RTCA (Real-Time Cell Analyzer) DP (Dual Plate) instrument according to manufacturers’ instructions (Roche Applied Science, Mannheim, Germany and ACEA Biosciences, San Diego, CA). The RTCA DP Instrument includes three main components: (i) RTCA DP Analyzer, which is placed inside a humidified incubator maintained at 37°C and 5% CO_2_, (ii) RTCA Control Unit with RTCA Software preinstalled, and (iii) E-Plate 16 for proliferation or CIM-plate 16 for migration and invasion assays. First, the optimal seeding number for each cell line (B-T549, MDA-MB-231, HCC-1143 and HCC-1937) was determined by cell titration and growth experiments. After seeding the respective number of cells/well (BT-549: 10,000 cells/well, MDA-MB-231: 20,000 cells/well, HCC-1143: 5,000 cells/well, and HCC-1937: 12,500 cells/well), the cells were automatically monitored every 15 minutes. Cells were treated with the compounds about four hours after seeding, when the cells were in the log growth phase. For cell proliferation assay in each cell line, cells were treated with DMSO as the vehicle or different concentrations of each Wnt inhibitor: iCRT-3 (25, 50, 75 μM), iCRT-5 (50, 100, 200 μM), iCRT-14 (10, 25, 50 μM), IWP-4 (1, 2.5, 5 μM), and XAV-939 (5, 10 μM). For cell proliferation, migration and invasion assays in BT549 cells with SOX4 knockdown, cells were treated with DMSO or 25 μM iCRT-3. The upper chamber of CIM-plate 16 was coated with Matrigel (1:40 dilution) for cell invasion assay. In addition, cell proliferation was measured in BT-549 cells with SOX4 knockdown that were treated with 50 μM genistein for six days, and 25 μM iCRT-3 at the time of the experiment. Each sample was assayed in triplicate, and three independent experiments were performed. Cell proliferation assays were run for 48 hours, and cell migration and invasion experiments for 24 hours. Cell index value, which is used to measure the relative change in electrical impedance to represent cell morphology, adhesion or viability, was calculated for each sample by the RTCA Software Package 1.2.

### Cell viability assay

Cells were seeded at 20,000 cells/well into 96-well plates. After overnight incubation, cells were treated with DMSO or each Wnt inhibitor (iCRT-3, 75 μM; iCRT-5, 200 μM; iCRT-14, 50 μM; IWP-4, 5 μM and XAV-939, 10 μM) for 48 hours. Cell viability was determined using the Cell Titer-Glo luminescent cell viability assay kit (Promega) according to the manufacturer’s instructions. Luminescence was measured using FLUOstar microplate reader. All treatments were performed in triplicate, and each experiment was repeated three times.

### Statistical analysis

Data obtained from three independent experiments performed in triplicate were presented as mean ± SEM. Student’s *t*-test (two-tailed, equal variance) was used to determine significant differences between two groups of data*. p* values of <0.05 and <0.01 were considered as statistically significant, and are indicated by asterisks (* and **, respectively).

### Bioinformatics meta-analysis

Gene expression data was downloaded from the Gene Expression Omnibus (GEO) repository using series accession GSE12790 derived from two studies of breast cancer cell lines [38,39]. Data was also obtained from the Cancer Cell Line Encyclopedia (CCLE) [40]. For the GSE12790 dataset, 43 luminal breast cancer cell lines were compared to 12 TNBC cell lines of mesenchymal, mesenchymal stem-like, or basal-like 2 subtypes of TNBC. For the CCLE dataset 22 luminal cell lines were compared to 21 TNBC cell lines. Differentially expressed genes were identified by Significance Analysis of Microarrays [41] with a false discovery rate of 5%, and pathway enrichment was determined by Ingenuity Pathway Analysis.

## Results

### Wnt signaling pathway is activated in TNBC cells

Previous studies have shown that Wnt pathway genes are upregulated in TNBC tumors [10]. To verify these earlier studies, we performed pathway enrichment analyses on two independent datasets. The first dataset (GSE12790) derived from two studies of breast cancer cell lines [38,39] included microarray data from 43 luminal breast cancer cell lines and 12 TNBC cell lines of mesenchymal, mesenchymal stem-like, or basal-like 2 subtypes. The second dataset included microarray data from the Cancer Cell Line Encyclopedia (CCLE) [40] with microarray data from 22 luminal breast cancer cell lines and 21 TNBC cell lines. For both the GSE12790 dataset and the CCLE dataset, Wnt-pathway genes were strongly enriched (p = 8.5e-6 and p = 3.0e-4, respectively). Genes differentially regulated in TNBC cell lines in both analyses included CD44, CDH1, DKK3, FZD7, SFRP1, SOX9, TGFB2, TLE4, WNT10A, and WNT5B (see Additional file 1: Table S1 and Additional file 2: Figure S1).

**Figure 1:**
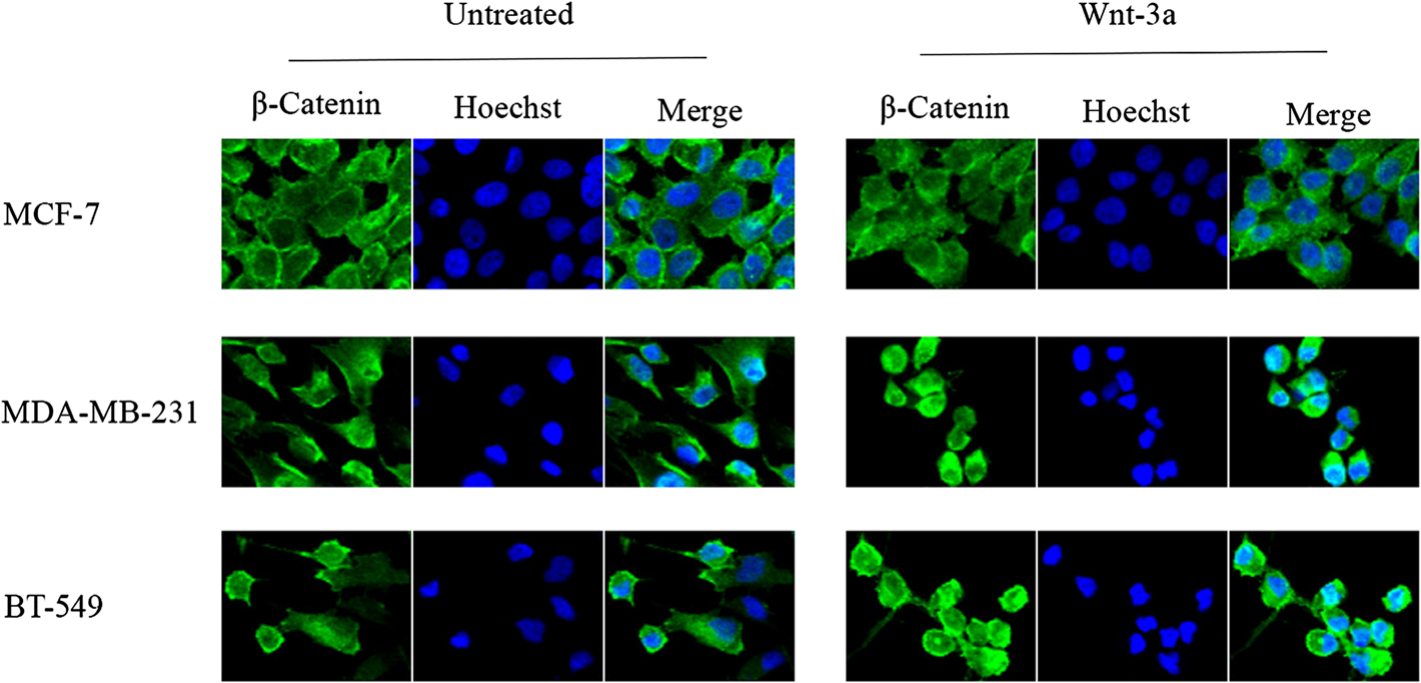
**Wnt signaling pathway is activated in TNBC cells.** Subcellular localization of β-catenin in TNBC cells treated with or without human recombinant Wnt-3a (200 ng/ml) for 4 hours was examined using confocal microscopy. Immunofluorescence staining of β-catenin (green) showed cytoplasmic localization in MCF-7 cell line, and was both nuclear and cytoplasmic in TNBC cell lines, MDA-MB-231 and BT-549. Treatment with Wnt-3a resulted in increased nuclear localization of β-catenin in MDA-MB-231 and BT-549 cells. Nuclei were counterstained with Hoechst 33342 (blue). Total magnification was 200×, and the images were zoomed in 500%.

**Figure 2:**
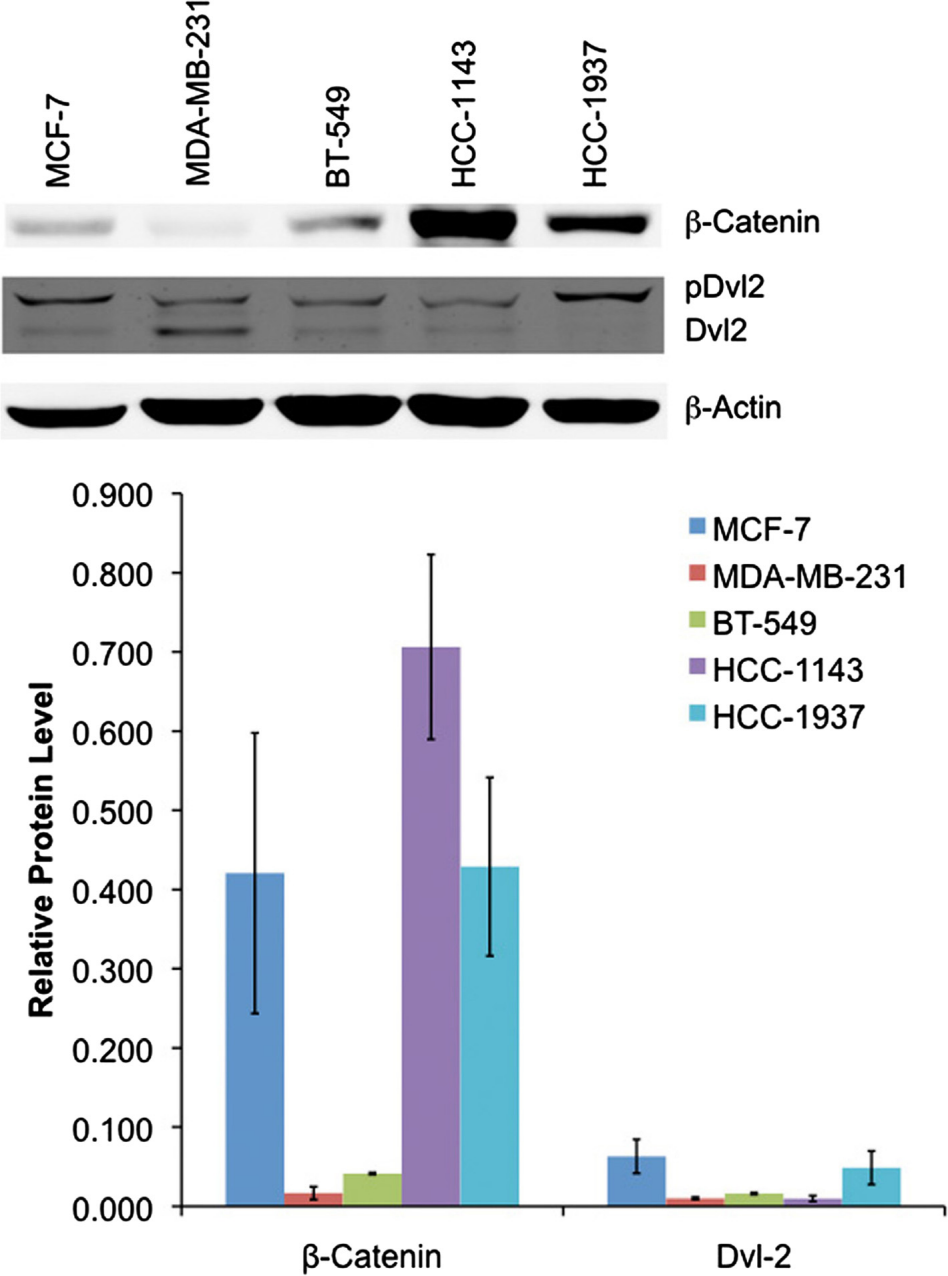
**Relative expression levels of active β-catenin and phosphorylated Dvl-2 in non-TNBC (MCF-7) and TNBC (MDA-MB-231, BT-549, HCC-1143 and HCC-1937) cell lines.** Whole cell lysates were prepared, and analyzed for protein expression using Western blotting. β-actin was used as normalization control. Data represent mean ± SEM of three independent experiments.

This prompted us to first assess whether the Wnt pathway was active in human TNBC cell lines by confocal microscopy. We selected two mesenchymal subtype TNBC cell lines (BT-549 and MDA-MB-231), and two basal-like 2 subtype TNBC cell lines (HCC-1143 and HCC-1937), as well as the non-TNBC ER^+^ control cell line MCF-7. Immunofluorescence staining of β-catenin showed both nuclear and cytoplasmic localization in TNBC cell lines whereas β-catenin was observed only in the cytoplasm of MCF-7 cells (Figure 1; see Additional file 3: Figure S2). We further explored the subcellular localization of β-catenin by treating the cells with Wnt-3a ligand (100 ng/ml) for four hours. We observed increased nuclear localization of β-catenin in MDA-MB-231 and BT-549 cells treated with Wnt-3a but not in MCF-7 cells, suggesting responsiveness of the canonical Wnt pathway in TNBC cells. To evaluate Wnt pathway activation, the expression levels of active β-catenin and phosphorylated Dvl-2 were examined in TNBC and non-TNBC cell lines by Western blot analysis (Figure 2). Active β-catenin was detected in both mesenchymal and basal-like subtypes, although the levels were much higher in basal-like cells than in mesenchymal cells. Activated β-catenin was lower in non-TNBC MCF-7 cells than in basal-like TNBC cells, but surprisingly was higher than in mesenchymal TNBC cells. Wnt-induced phosphorylation of Dvl-2 associated with mobility shift was differentially observed in all cell lines, with the highest ratio in HCC-1937 and the lowest in MDA-MB-231 cells.

### iCRT-3 effectively inhibits cell proliferation in TNBC cells

**Figure 3:**
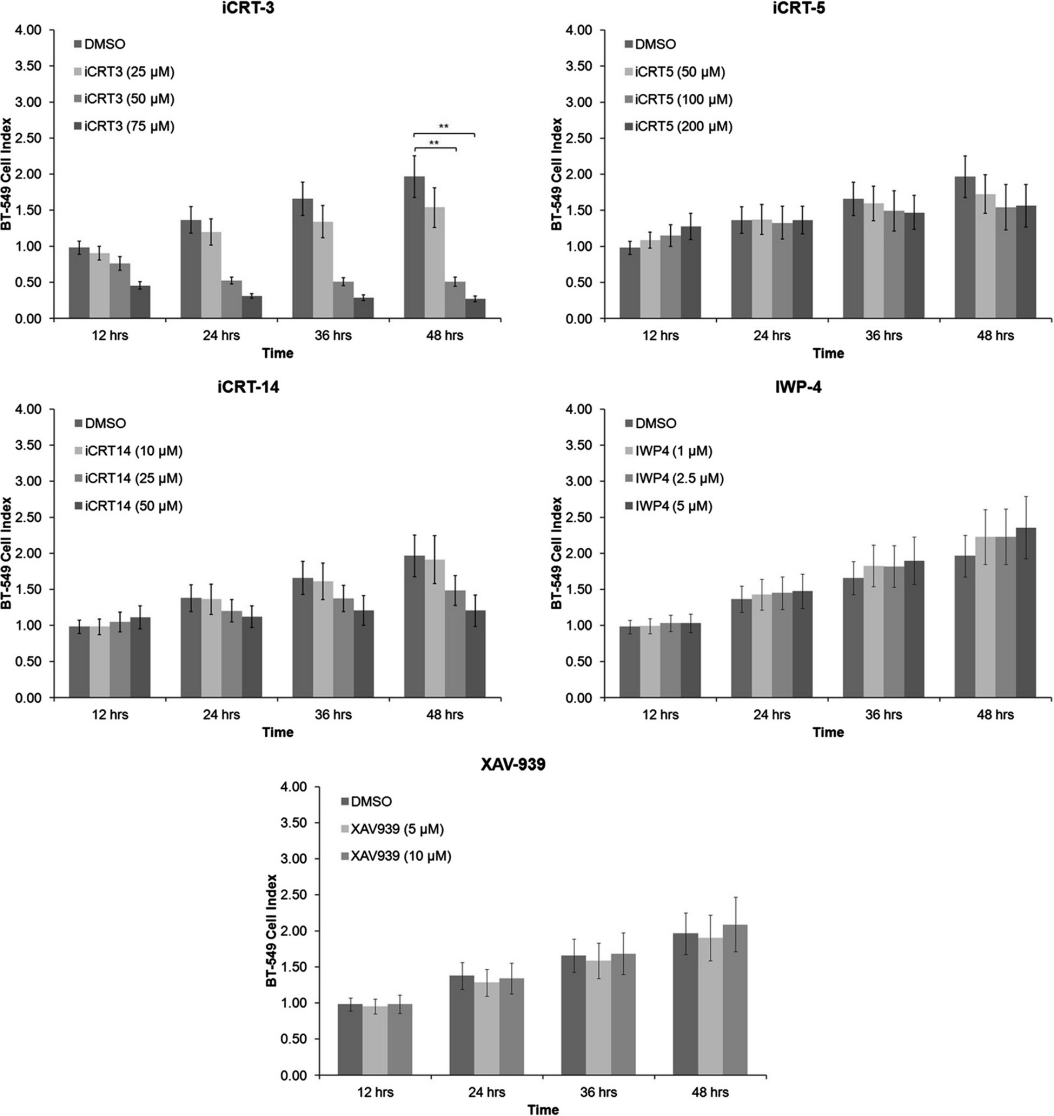
**iCRT-3 effectively inhibits cell proliferation in BT-549 cells in a dose- and time-dependent manner.** Cells were treated with vehicle (DMSO) or each of five Wnt inhibitors (iCRT-3, iCRT-5, iCRT-14, IWP-4, and XAV-939) at the indicated concentrations. Cell index values were continuously measured for 48 hours at intervals of 15 minutes using an xCELLigence instrument. Data represent mean ± SEM of three independent experiments (**p < 0.01).

To investigate the effectiveness of five different compounds targeting the Wnt pathway in breast cancer cells, we first tested the inhibitory effects iCRT-3, iCRT-5, iCRT-14, IWP-4, and XAV-939 on cell proliferation in BT-549, MDA-MB-231, HCC-1143 and HCC-1937 cell lines using the xCELLigence system that allows continuous and quantitative monitoring of cell status in real-time. Cells were treated with increasing concentrations of each compound and assayed for 48 hours. The concentration range for treatment with each inhibitor was determined based on previous studies (34–36). This analysis showed that each compound induced differential effects on proliferation of these TNBC cells in a dose- and time-dependent manner (Figure 3; see Additional file 4: Figure S3, Additional file 5: Figure S4 and Additional file 6: Figure S5). These findings were confirmed using an alternative cell viability assay, the Cell Titer-Glo luminescent cell viability assay (see Additional file 7: Figure S6). Taken together, these data indicated that iCRT-3 was the most effective compound that we tested for inhibiting proliferation in all of these TNBC cells.

### Wnt pathway is antagonized by iCRT-3 in BT-549 cells

**Figure 4:**
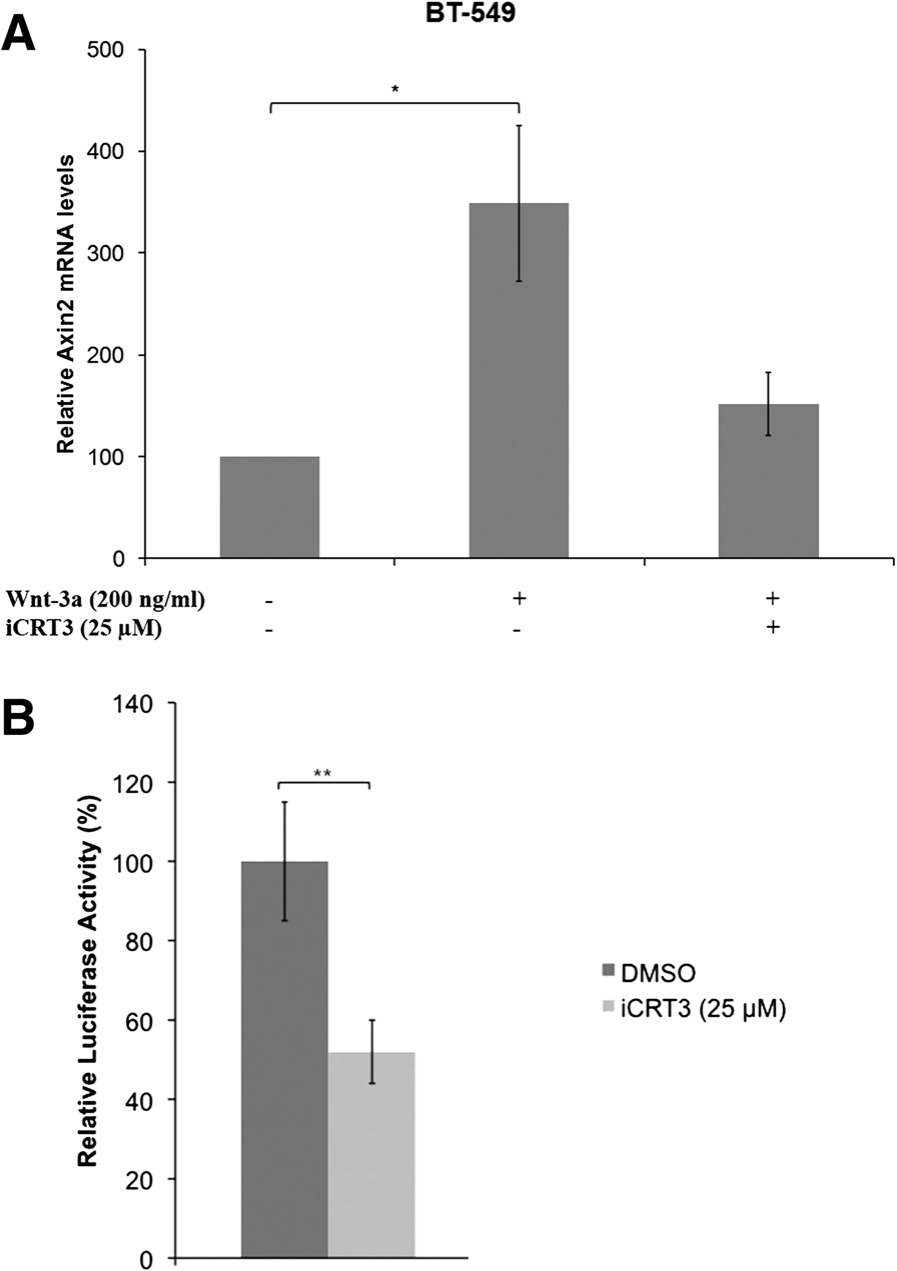
**iCRT-3 inhibits Wnt signaling in TNBC cells. (A)** Wnt pathway is antagonized by iCRT-3. BT-549 cells were serum-starved for 24 hours, and treated with Wnt-3a (200 ng/ml) and/or iCRT-3 (25 μM) for 4 hours. Total RNA was prepared, and assessed for Axin2 expression using quantitative real-time RT-PCR. β-actin was used as normalization control. Data represent mean ± SEM of three independent experiments (*p < 0.05). **(B)** iCRT-3 significantly inhibits transcriptional activity of β-catenin in BT-549 cells. BT-549 cells were transiently transfected with TOP-FLASH firefly luciferase reporter vector and *Renilla* luciferase vector. After 24 hour-transfection, cells were treated with DMSO or 25 μM iCRT-3 for 48 hours. Luciferase activities were measured using Dual-Luciferase Reporter Assay System. The relative luciferase activity was calculated by firefly luciferase activity/Renilla luciferase activity. Data represent mean ± SEM of three independent experiments (**p < 0.01).

To evaluate whether the inhibitory effects of iCRT-3 are mediated through canonical Wnt signaling in TNBC, BT-549 cells were serum-starved for 24 hours, and then treated with Wnt-3a (200 ng/ml) and/or iCRT-3 (25 μM) for four hours. Quantitative real-time RT-PCR analysis of Axin2 in these cells showed that Wnt pathway is significantly activated and iCRT-3 efficiently blocked the expression of Axin2, which is a Wnt-induced target gene (Figure 4A). However, none of the other Wnt inhibitors had inhibitory effect on Axin2 expression (see Additional file 8: Figure S7). Previous studies have reported that iCRT-3 efficiently blocks the transcriptional function of β-catenin [22,34]. To assess the effect of iCRT-3 treatment on transcriptional activity of β-catenin in BT-549 cells, dual luciferase assay was performed using the TOP-FLASH reporter vector. Cells were transfected with TOP-FLASH reporter and *Renilla* control vectors. After 24 hour-transfection, cells were treated with DMSO or 25 μM iCRT-3, and luciferase activity was measured at 48 hours post-treatment. iCRT-3 treatment of BT-549 cells resulted in significant decrease in transcriptional activity of β-catenin, suggesting that iCRT-3 inhibits the canonical Wnt pathway (Figure 4B). These data demonstrate that iCRT-3 antagonizes Wnt pathway signaling.

### SOX4 knockdown synergizes with iCRT-3 to induce apoptosis in BT-549 cells

**Figure 5:**
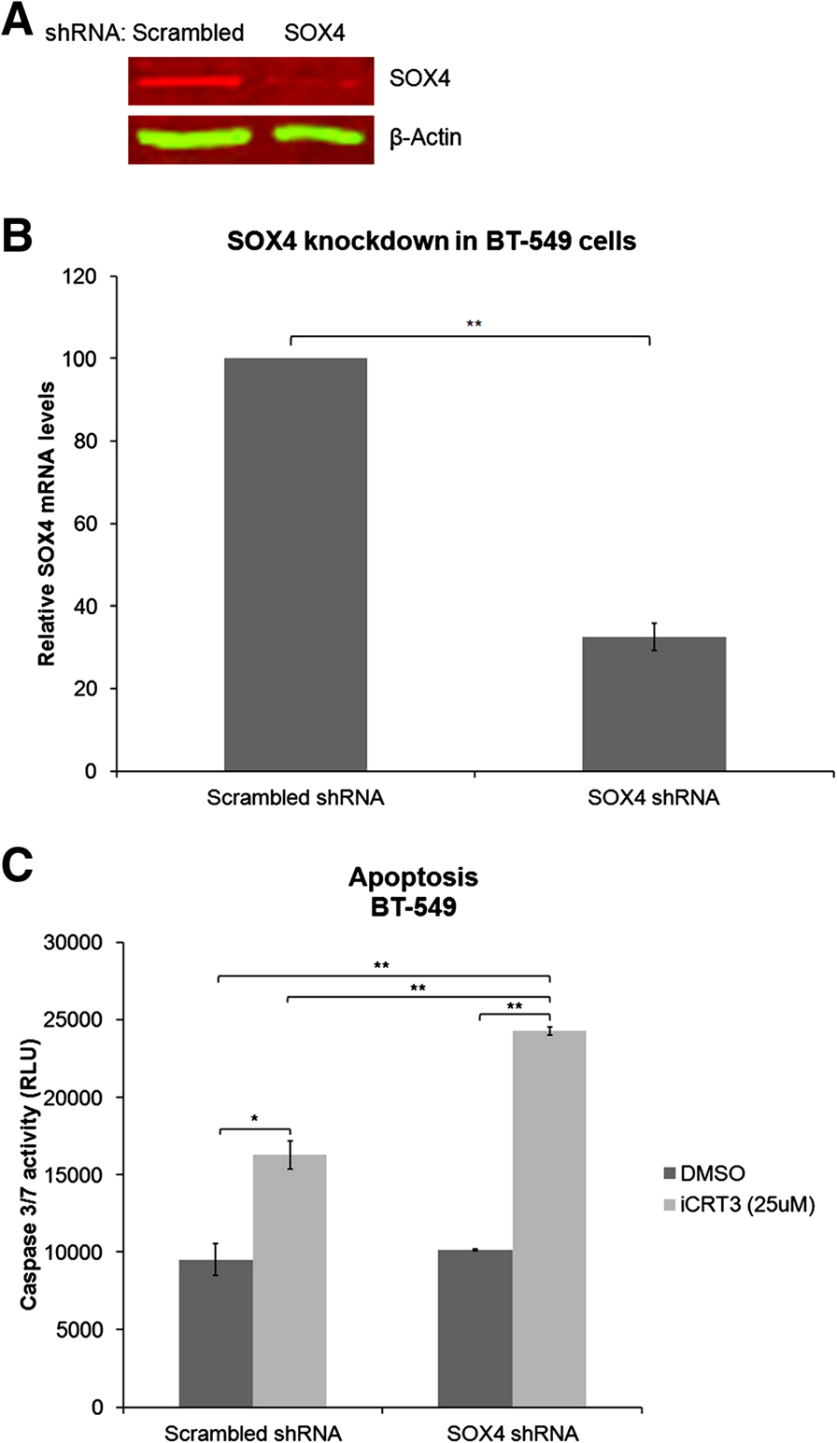
**SOX4 knockdown enhances the effects of iCRT-3 on apoptosis in BT-549 cells.** BT-549 cells were transduced with scrambled shRNA or SOX4 shRNA lentiviral particles. For stable knockdown, cells were kept under puromycin selection for two weeks. Whole cell lysates and total RNA were prepared, and assessed for SOX4 expression using **(A)** Western blotting and **(B)** quantitative real-time RT-PCR. β-actin was used as normalization control. Data represent mean ± SEM of three independent experiments (**p < 0.01). **(C)** Apoptosis levels were quantitated by Caspase-Glo 3/7 assay. Data represent mean ± SEM of three independent experiments (**p < 0.01, *p < 0.05).

Previous studies have shown that the oncogenic SOX4 transcription factor plays an important role in Wnt signaling pathways in many cancers including TNBC [10,18,19]. Therefore, we hypothesized that knockdown of SOX4 could inhibit cell viability and induce apoptosis in TNBC cells. To test our hypothesis, we first transduced the BT-549, MDA-MB-231, HCC-1143 and HCC-1937 cells with scrambled or SOX4 shRNA lentiviral particles. However, generation of stable SOX4 knockdown was successful only in BT-549 cells, possibly because SOX4 knockdown may be lethal to the other lines tested. Western blotting and quantitative real-time RT-PCR analyses demonstrated that the expression of SOX4 protein in BT-549 cells transduced with SOX4 shRNA was significantly decreased in comparison with that of cells transduced with scrambled shRNA, verifying that the expression of SOX4 was successfully knocked down in BT-549 cells (Figure 5A and B). Moreover, Caspase 3/7 activities showed that while knockdown of SOX4 alone did not enhance apoptosis of these cells (Figure 5C), combined treatment of iCRT-3 with SOX4 knockdown has a synergistic effect in inducing apoptosis.

### SOX4 knockdown inhibits cell proliferation and migration of BT-549 cells, and cooperates with iCRT-3 to inhibit proliferation

**Figure 6:**
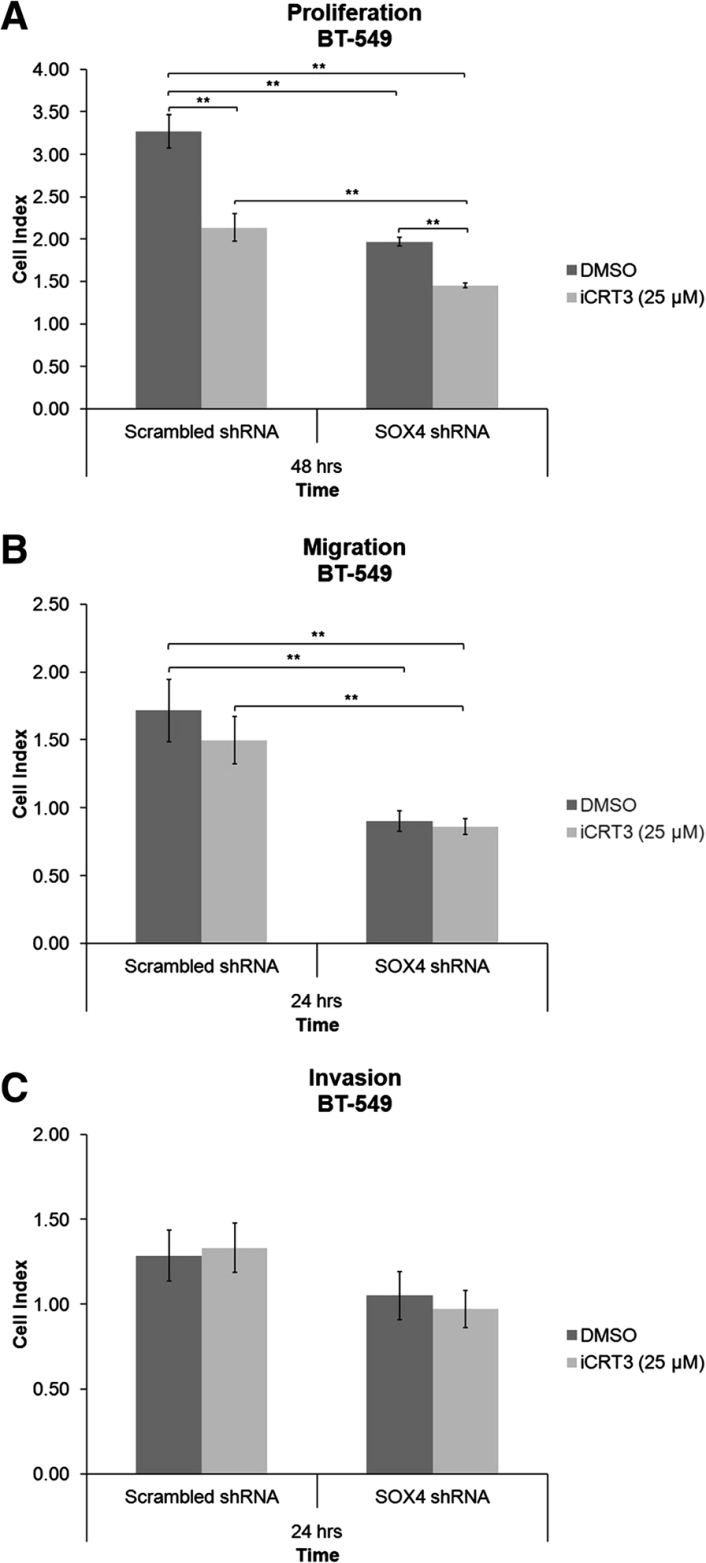
**SOX4 knockdown inhibits proliferation, migration and invasion of BT-549 cells, and cooperates with iCRT-3 to inhibit proliferation.** Cells that were transduced with scrambled shRNA or SOX4 shRNA lentiviral particles were treated with vehicle (DMSO) or iCRT-3 (25 μM)**,** and cell index measurements were continuously taken for 48 hours in **(A)** proliferation assay, and 24 hours in **(B)** migration and **(C)** invasion assays using an xCELLigence instrument. Data represent mean ± SEM of three independent experiments (**p < 0.01).

To characterize the mechanism underlying the synergism between SOX4 knockdown and iCRT-3 treatment, we next assessed the effect of SOX4 knockdown in combination with iCRT-3 treatment on cell proliferation, migration and invasion in BT-549 cells using the xCELLigence system. Cells that were transduced with scrambled or SOX4 shRNAs were treated with DMSO or 25 μM iCRT-3, and monitored continuously for 48 hours in a proliferation assay, and for 24 hours in migration and invasion assays. As shown in Figure 6, SOX4 knockdown inhibited cell proliferation and migration. Effects on invasion were not statistically significant, although the trend was towards lower invasion with SOX4 knockdown. Combination treatment of iCRT-3 with SOX4 knockdown induced further decrease in cell proliferation, but had no increased effect over SOX4 knockdown in cell migration or invasion.

**Figure 7:**
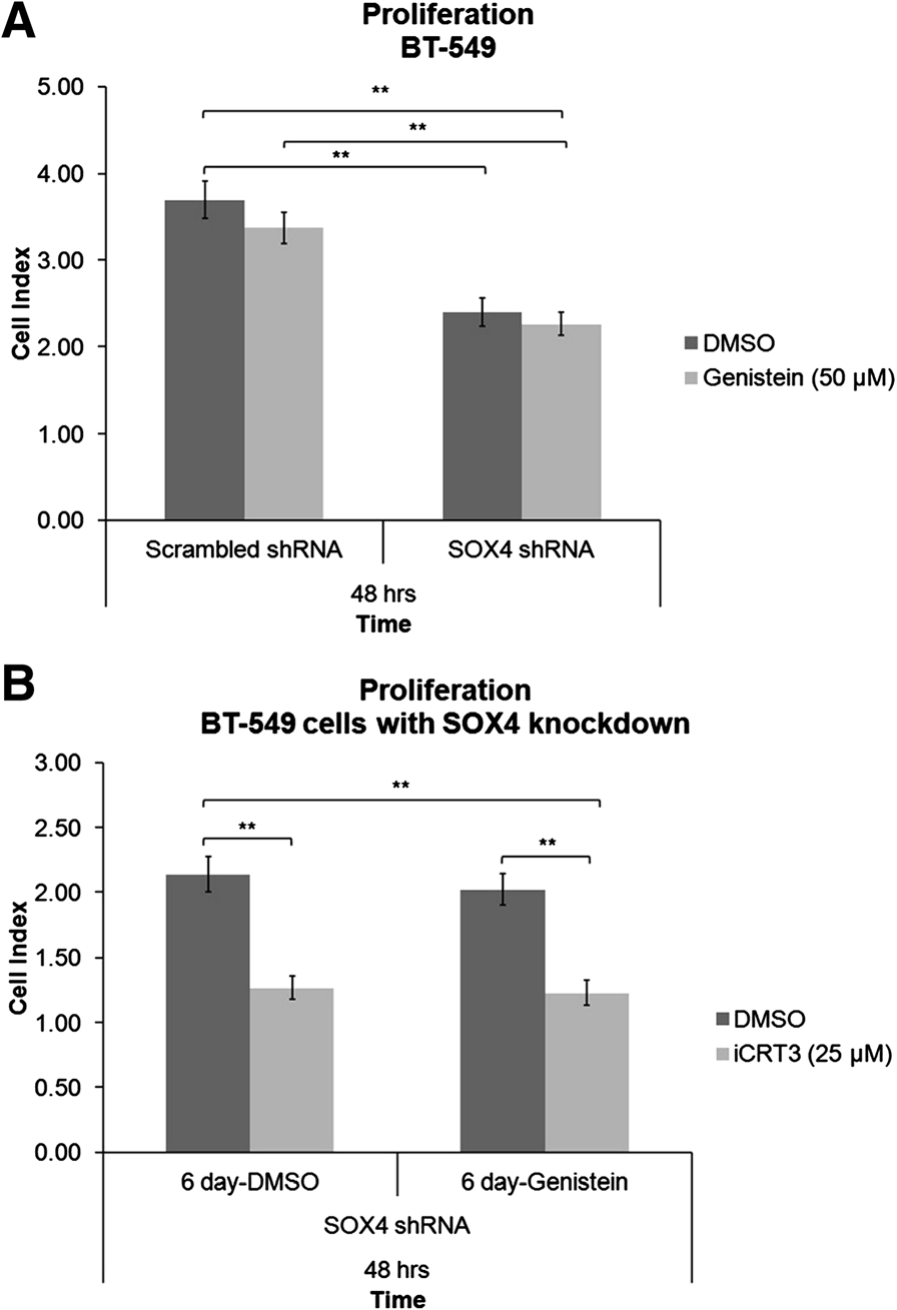
**Genistein has no inhibitory effect on cell proliferation in BT-549 cells. (A)** Cells that were transduced with scrambled shRNA or SOX4 shRNA lentiviral particles were treated with vehicle (DMSO) or genistein (50 μM) for six days. **(B)** These cells were further treated with iCRT-3 (25 μM) at the time of the proliferation assay, and cell index measurements were taken for 48 hours using an xCELLigence instrument. Data represent mean ± SEM of three independent experiments (**p < 0.01).

Genistein has been shown to have anticancer effects through targeting multiple signaling pathways, including Wnt signaling [31,32]. Therefore, we tested the effect of genistein in combination with SOX4 knockdown and/or iCRT-3 treatment on proliferation in BT-549 cells. Cells that were transduced with scrambled or SOX4 shRNAs were treated with DMSO or 50 μM genistein for six days. Cells were then treated with 25 μM iCRT-3, and monitored for 48 hours (Figure 7). Genistein treatment did not enhance the inhibitory effects of either SOX4 knockdown or iCRT-3 treatment on cell proliferation in BT-549 cells.

## Discussion

Due to the lack of hormone receptors and HER2 overexpression in patients with TNBC, hormonal therapies and HER2-targeted agents are ineffective, and chemotherapy is the main current systemic treatment in this subtype of breast cancer [9]. Although they are sometimes responsive to neoadjuvant chemotherapy, TNBC patients have a higher rate of relapse with distant metastases and a poorer prognosis than women with other breast cancer subtypes [3,4,6-8]. These factors highlight the urgent need to develop more effective and targeted therapy options for patients with TNBC. Aberrant activation of Wnt signaling, which plays a key role in the regulation of cell growth, development and differentiation, has been associated with many cancer types including colorectal, prostate, liver, and breast cancers [16-19]. Thus, inhibition of Wnt signaling could potentially be an effective approach to the treatment of many types of human cancers.

In the present study, we investigated, for the first time, the effects of five different small-molecule inhibitors that target different components of the Wnt signaling pathway on cell proliferation in four TNBC cell lines. These representative cells belong to mesenchymal or basal-like 2 subtypes of TNBC in which Wnt pathway-associated genes are specifically overexpressed. While mesenchymal TNBC cells (MDA-MB-231 and BT-549) responded to Wnt-3a treatment (Figure 1), basal-like 2 TNBC cells (HCC-1143 and HCC-1937) did not even though they did respond to iCRT-3 treatment. One hypothesis to explain this observation is that basal-like 2 TNBC cells may respond to different Wnt family ligands such as Wnt-5a, Wnt-5b, or Wnt-10a. The small compound Wnt inhibitors that we utilized in this study included (i) iCRT-3, iCRT-5 and iCRT-14 which inhibit catenin responsive transcription (CRT) [35], (ii) IWP-4, an inhibitor of Wnt production (IWP) that targets the acetyltransferase porcupine [37], and (iii) XAV-939, which induces β-catenin degradation by stabilizing axin through inhibition of poly-ADP-ribosylating enzymes tankyrase 1 and tankyrase 2 [36]. Our findings indicated that each inhibitor had differential effects on proliferation of each cell line. The mesenchymal MDA-MB-231 and BT-549 cells were more sensitive than the basal-like HCC-1143 and HCC-1937 cells. We speculate that these differentials are due to the different levels of basal Wnt activation and different genetic backgrounds of the cell lines that could provide for different mechanisms of constitutive Wnt activation. Surprisingly, the cell lines with the lower constitutive Wnt activity are more sensitive to the inhibitors. It is not clear why this is the case, but we can speculate that the inhibitors may be less effective when Wnt activation levels are above a certain threshold. Alternatively, the less sensitive cell lines may have activation of additional anti-apoptotic, pro-survival pathways that confer resistance to Wnt pathway inhibition.

Our data also demonstrated that of these five compounds, iCRT-3 was the most effective and consistent one in inhibiting cell proliferation in all TNBC cell lines tested. Because iCRTs, IWP-4 and XAV-939 inhibit Wnt signals through different mechanisms, the effectiveness of each inhibitor would be expected to vary in these TNBC cell lines depending on the genetic changes they have. Previous reports have shown that IWP-4 and XAV-939 are effective in cells which have loss of APC tumor suppressor function, and this effectiveness could be explained by the rate-limiting role that Axin proteins play in canonical Wnt pathway. Our finding that there was no significant inhibitory effect of IWP-4 and XAV-939 on proliferation of the TNBC cell lines examined in this study may correlate with the fact that none of these TNBC cell lines has a mutation in APC gene. In addition, iCRT-3 resulted in increased apoptosis in BT-549 cells, whereas knockdown of SOX4 expression did not have a significant effect on apoptosis. Combined treatment of SOX4 knockdown with iCRT-3 synergistically induced apoptosis in BT-549 cells. It is noteworthy that in the combination experiments, we treated the cells with a suboptimal concentration of 25 μM iCRT-3 in order to enable detection of synergistic effects of combination treatments. Luciferase reporter assays showed that iCRT-3 significantly antagonized canonical Wnt pathway in BT-549 cells, consistent with our finding that expression of Axin2 was suppressed by iCRT-3 in these cells. Another important finding in this study is that knockdown of SOX4 in BT-549 cells had inhibitory effects on cell proliferation and migration. Moreover, iCRT-3 treatment enhanced SOX4 knockdown-induced inhibition of cell proliferation, but did not have an additive effect over SOX4 knockdown on migration and invasion of BT-549 cells.

## Conclusions

We show in this study that iCRT-3 treatment inhibits proliferation and induces apoptosis, whereas SOX4 knockdown effectively inhibits cell proliferation and migration, suggesting potential therapeutic roles for iCRT-3 and SOX4 in targeting TNBC. These findings highlight the importance of the Wnt signaling cascade in TNBC progression, and provide a strong rationale for future *in vivo* studies of these agents in TNBC. Further investigation of the molecular mechanisms of TNBC will provide a better understanding of the pathogenesis, and thus additional insight into the development of new, targeted therapeutics for TNBC.

## Competing interests

Authors have no competing interests.

## Authors’ contributions

BB contributed experimental design, acquisition and analysis of data, interpretation of findings, and writing of the manuscript. OK contributed experimental design and editing of the manuscript. CSM contributed experimental design, analysis of data, interpretation of findings, and editing of the manuscript. All authors read and approved the final manuscript.

## Supplementary Material

Additional file 1: Table S1 List of genes differentially expressed in TNBC cell lines in microarray analyses for Wnt pathway genes. Click here for file

Additional file 2: Figure S1 Wnt pathway analysis of TNBC. Click here for file

Additional file 3: Figure S2 Subcellular localization of β-catenin in HCC-1143 and HCC-1937 cells treated with or without human recombinant Wnt-3a (200 ng/ml) for 4 hours was examined using confocal microscopy. Immunofluorescence staining of β-catenin (green) showed cytoplasmic localization in both cell lines. Treatment with Wnt-3a did not have an effect on subcellular localization of β-catenin in HCC-1143 and HCC-1937 cells. Nuclei were counterstained with Hoechst 33342 (blue). Total magnification was 200×, and the images were zoomed in 500%. Click here for file

Additional file 4: Figure S3 iCRT-3 effectively inhibits cell proliferation in HCC-1143 cells in a dose- and time-dependent manner. Cells were treated with vehicle (DMSO) or each of five Wnt inhibitors (iCRT-3, iCRT-5, iCRT-14, IWP-4, and XAV-939) at the indicated concentrations. Cell index values were continuously measured for 48 hours at intervals of 15 minutes using an xCELLigence instrument. Data represent mean ± SEM of three independent experiments (**p < 0.01). Click here for file

Additional file 5: Figure S4 iCRT-3 effectively inhibits cell proliferation in MDA-MB-231 cells in a dose- and time-dependent manner. Cells were treated with vehicle (DMSO) or each of five Wnt inhibitors (iCRT-3, iCRT-5, iCRT-14, IWP-4, and XAV-939) at the indicated concentrations. Cell index values were continuously measured for 48 hours at intervals of 15 minutes using an xCELLigence instrument. Data represent mean ± SEM of three independent experiments (**p < 0.01). Click here for file

Additional file 6: Figure S5 iCRT-3 effectively inhibits cell proliferation in HCC-1937 cells in a dose- and time-dependent manner. Cells were treated with vehicle (DMSO) or each of five Wnt inhibitors (iCRT-3, iCRT-5, iCRT-14, IWP-4, and XAV-939) at the indicated concentrations. Cell index values were continuously measured for 48 hours at intervals of 15 minutes using an xCELLigence instrument. Data represent mean ± SEM of three independent experiments (**p < 0.01). Click here for file

Additional file 7: Figure S6 iCRT-3 effectively inhibits cell proliferation in MDA-MB-231, BT-549, HCC-1143 and HCC1937 cells. Cells were treated with vehicle (DMSO) or each of five Wnt inhibitors (iCRT-3, iCRT-5, iCRT-14, IWP-4, and XAV-939) at the indicated concentrations. Cell viability was measured using Cell Titer-Glo luminescent cell viability assay. Data represent mean ± SEM of three independent experiments (**p < 0.01). Click here for file

Additional file 8: Figure S7 Wnt pathway is not antagonized by iCRT-5, iCRT-14, IWP-4 or XAV-939. BT-549 cells were serum-starved for 24 hours, and treated with Wnt-3a (200 ng/ml) and/or iCRT-5 (50 μM), iCRT-14 (10 μM), IWP-4 (1 μM) or XAV-939 (5 μM) for 4 hours. Total RNA was prepared, and assessed for Axin2 expression using quantitative real-time RT-PCR. β-actin was used as normalization control. Click here for file
