# Supplementary figures and images for: Wnt signaling blockage inhibits cell proliferation and migration, and induces apoptosis in triple-negative breast cancer cells
Source: J Transl Med. 2013 Nov 4;11:280. doi: 10.1186/1479-5876-11-280 (PMC4228255; doi:10.1186/1479-5876-11-280)

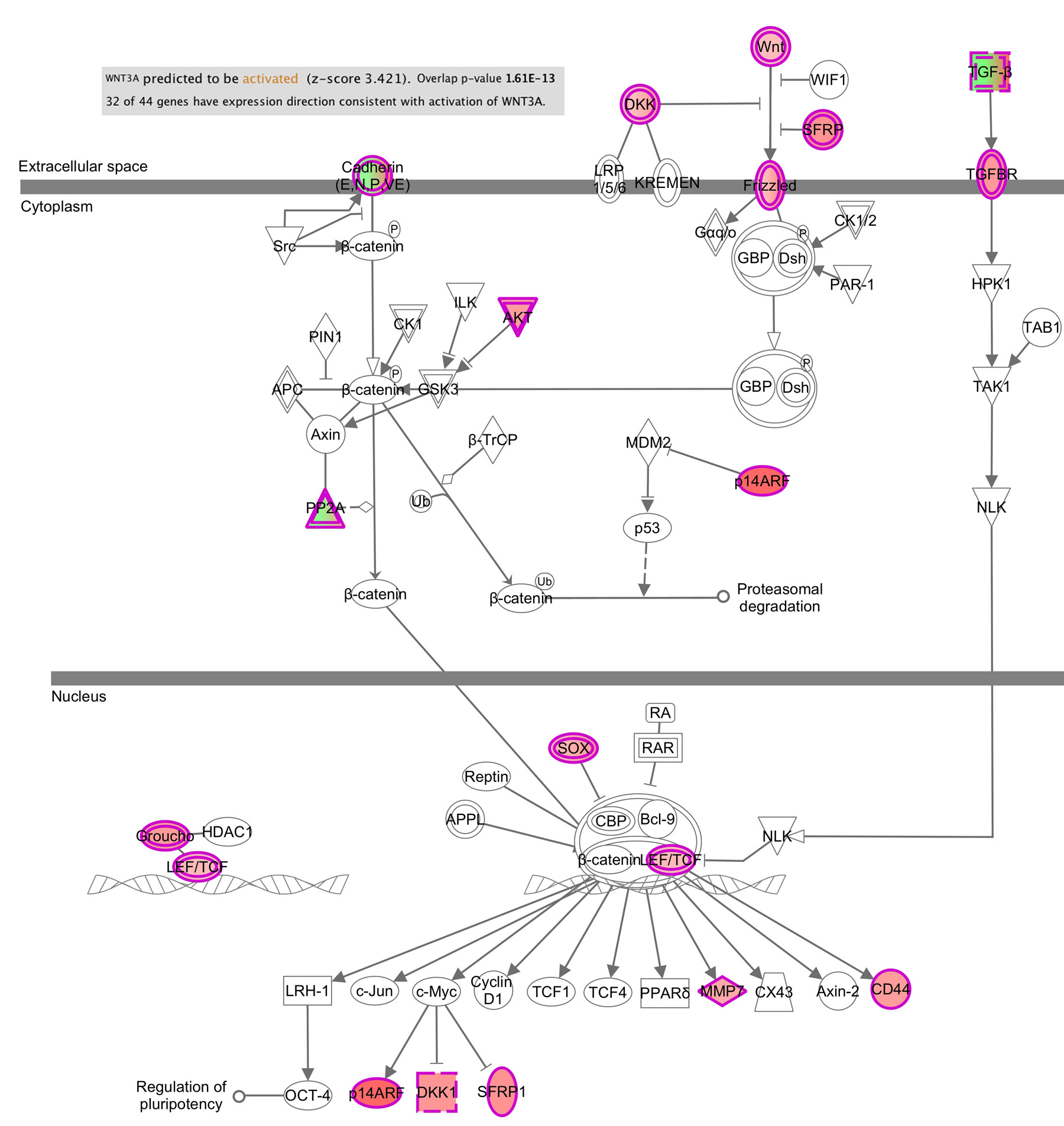

Supplement: Additional file 2: Figure S1 — Wnt pathway analysis of TNBC. [file 1479-5876-11-280-S2.jpeg]

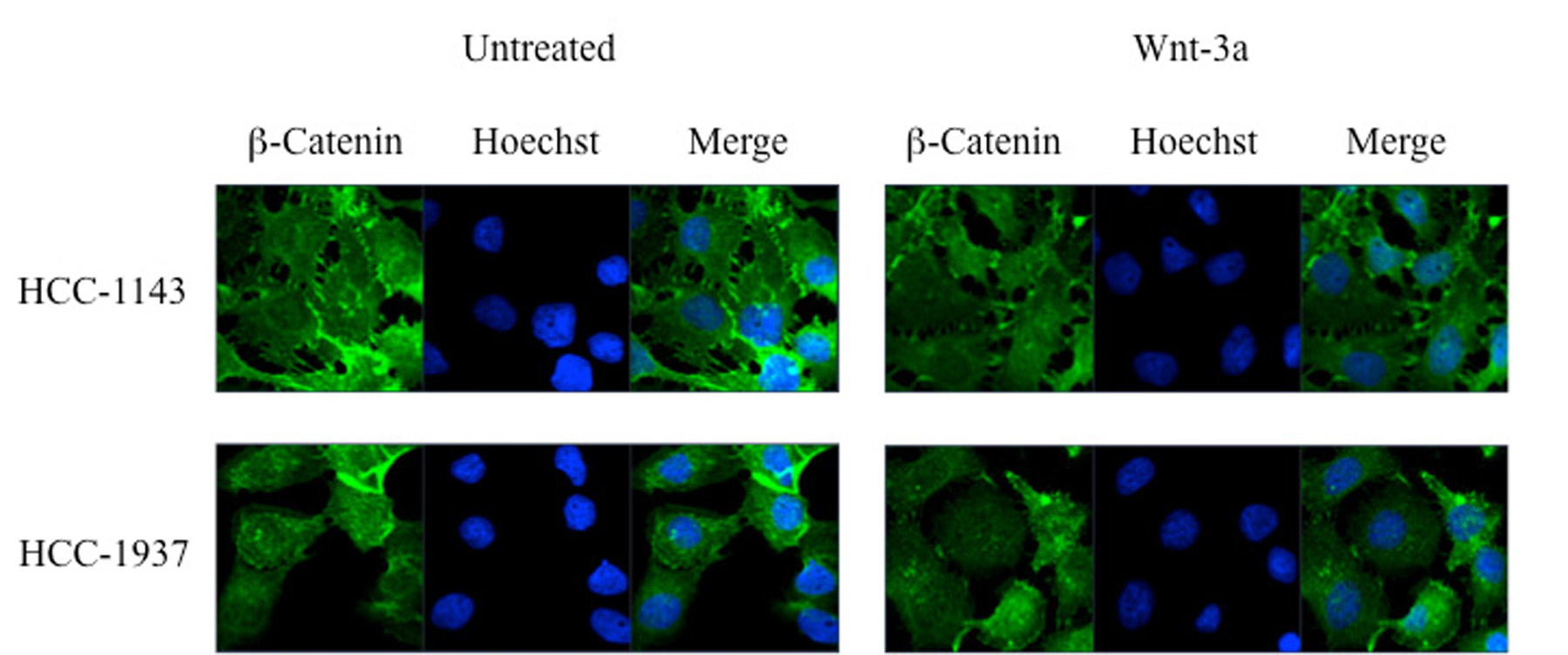

Supplement: Additional file 3: Figure S2 — Subcellular localization of β-catenin in HCC-1143 and HCC-1937 cells treated with or without human recombinant Wnt-3a (200 ng/ml) for 4 hours was examined using confocal microscopy. Immunofluorescence staining of β-catenin (green) showed cytoplasmic localization in both cell lines. Treatment with Wnt-3a did not have an effect on subcellular localization of β-catenin in HCC-1143 and HCC-1937 cells. Nuclei were counterstained with Hoechst 33342 (blue). Total magnification was 200×, and the images were zoomed in 500%. [file 1479-5876-11-280-S3.jpeg]

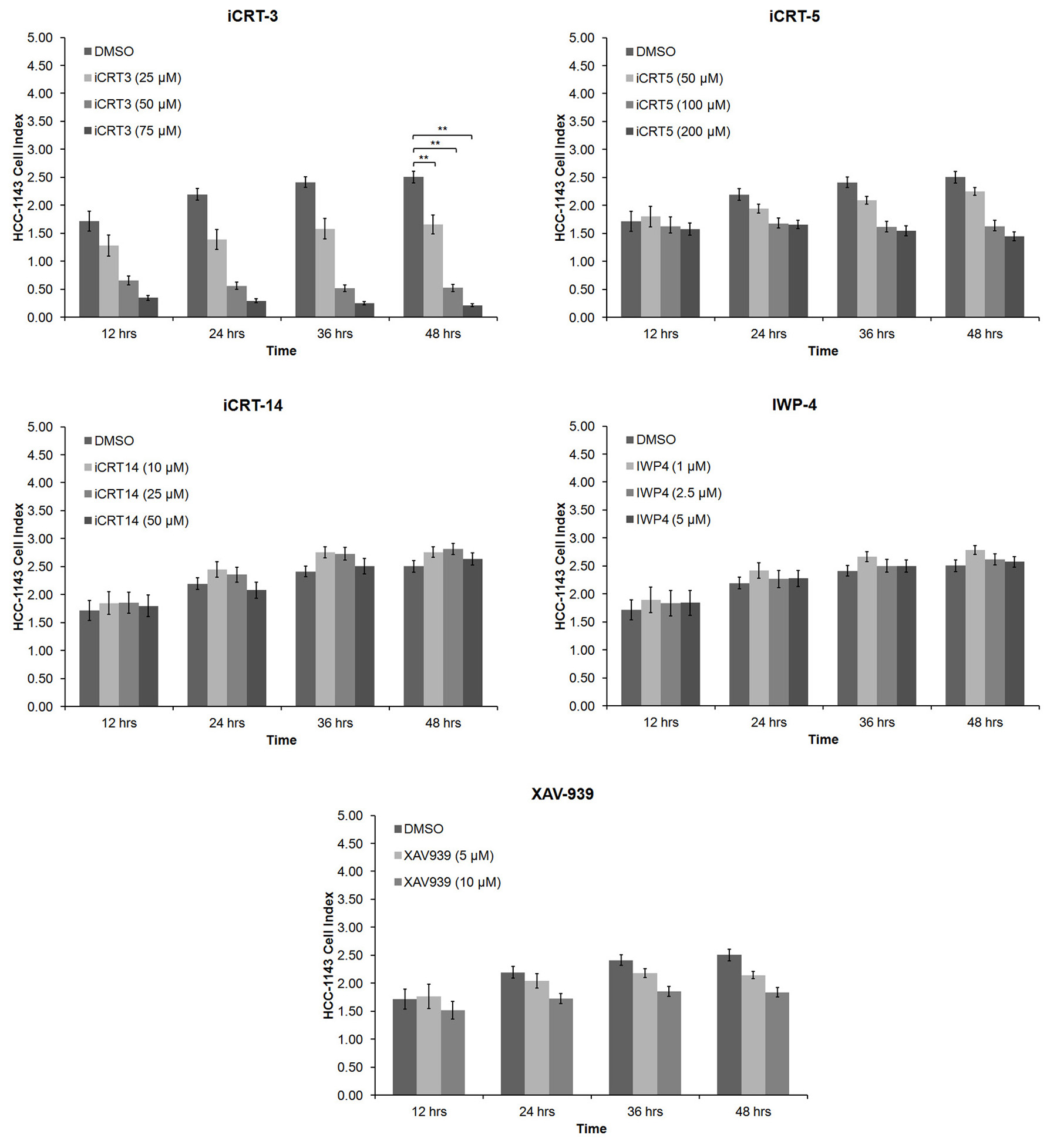

Supplement: Additional file 4: Figure S3 — iCRT-3 effectively inhibits cell proliferation in HCC-1143 cells in a dose- and time-dependent manner. Cells were treated with vehicle (DMSO) or each of five Wnt inhibitors (iCRT-3, iCRT-5, iCRT-14, IWP-4, and XAV-939) at the indicated concentrations. Cell index values were continuously measured for 48 hours at intervals of 15 minutes using an xCELLigence instrument. Data represent mean ± SEM of three independent experiments (**p < 0.01). [file 1479-5876-11-280-S4.jpeg]

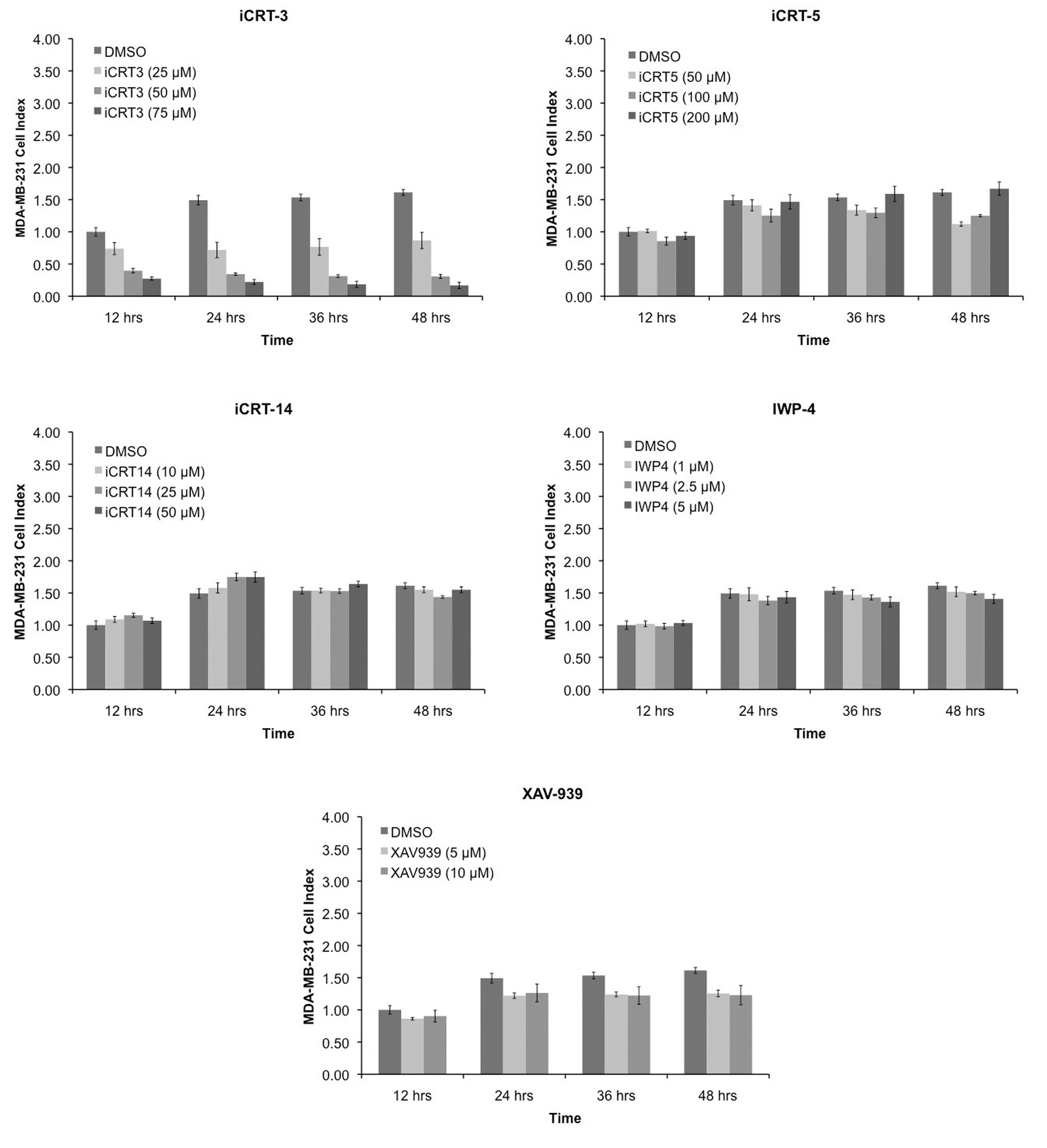

Supplement: Additional file 5: Figure S4 — iCRT-3 effectively inhibits cell proliferation in MDA-MB-231 cells in a dose- and time-dependent manner. Cells were treated with vehicle (DMSO) or each of five Wnt inhibitors (iCRT-3, iCRT-5, iCRT-14, IWP-4, and XAV-939) at the indicated concentrations. Cell index values were continuously measured for 48 hours at intervals of 15 minutes using an xCELLigence instrument. Data represent mean ± SEM of three independent experiments (**p < 0.01). [file 1479-5876-11-280-S5.jpeg]

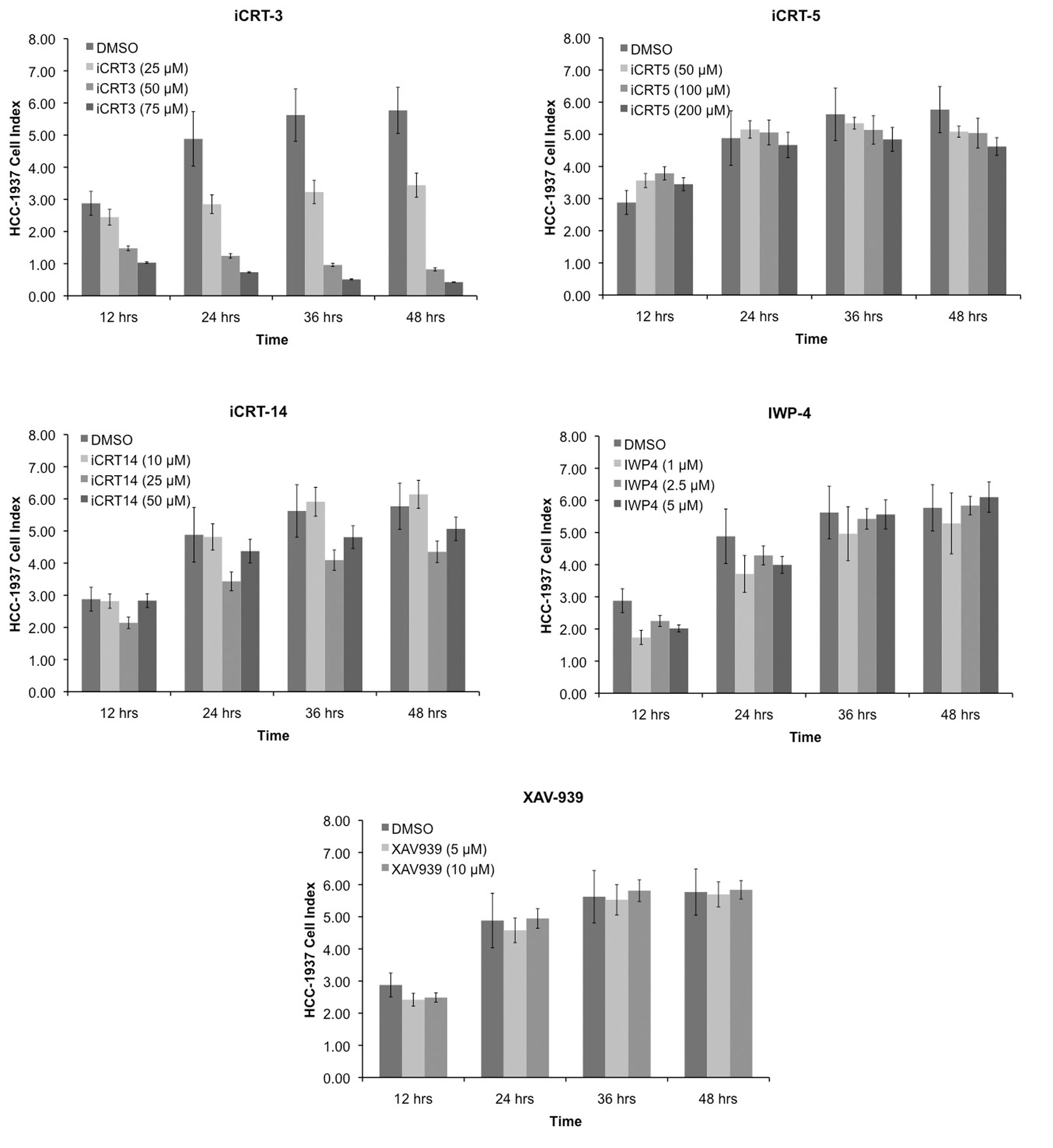

Supplement: Additional file 6: Figure S5 — iCRT-3 effectively inhibits cell proliferation in HCC-1937 cells in a dose- and time-dependent manner. Cells were treated with vehicle (DMSO) or each of five Wnt inhibitors (iCRT-3, iCRT-5, iCRT-14, IWP-4, and XAV-939) at the indicated concentrations. Cell index values were continuously measured for 48 hours at intervals of 15 minutes using an xCELLigence instrument. Data represent mean ± SEM of three independent experiments (**p < 0.01). [file 1479-5876-11-280-S6.jpeg]

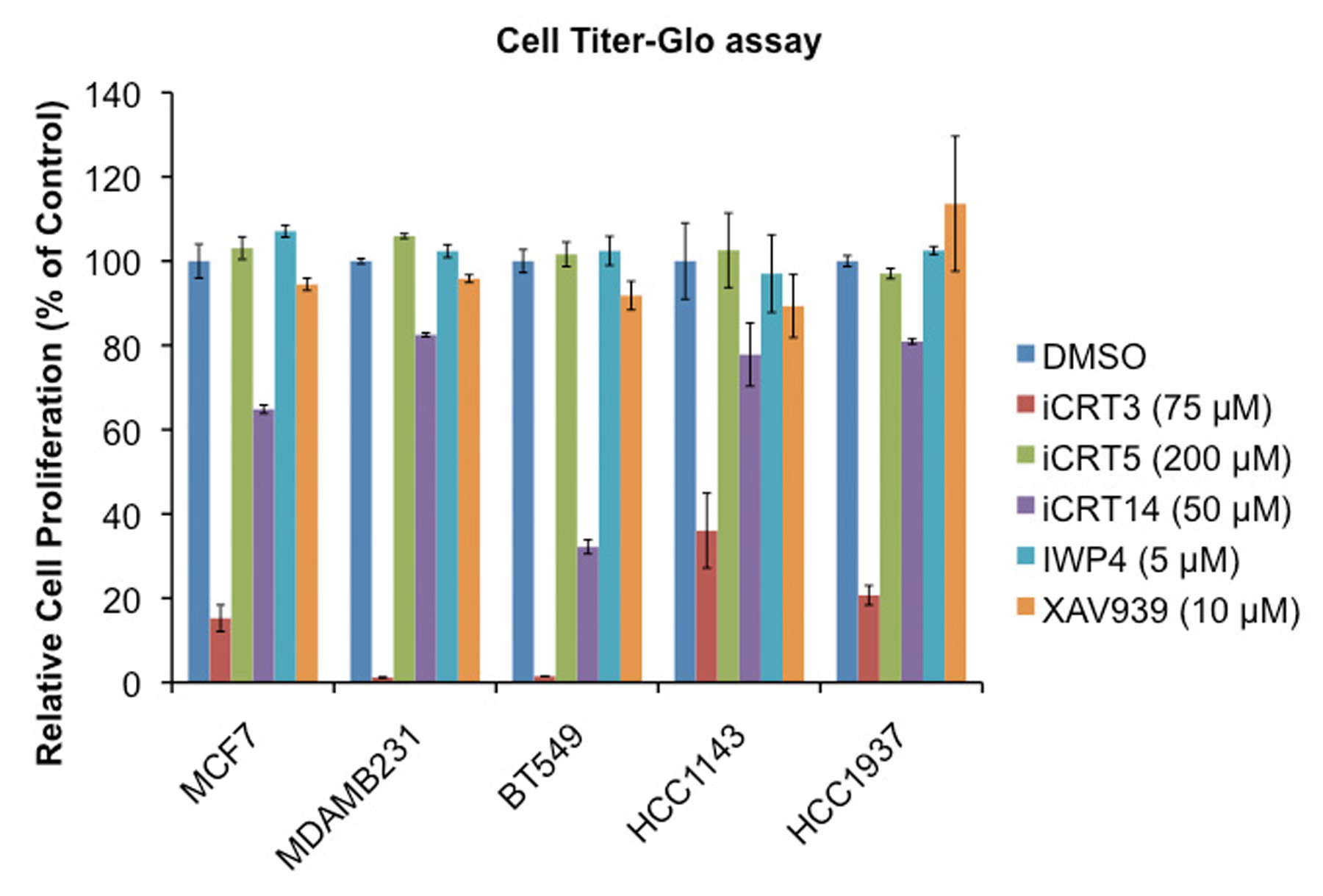

Supplement: Additional file 7: Figure S6 — iCRT-3 effectively inhibits cell proliferation in MDA-MB-231, BT-549, HCC-1143 and HCC1937 cells. Cells were treated with vehicle (DMSO) or each of five Wnt inhibitors (iCRT-3, iCRT-5, iCRT-14, IWP-4, and XAV-939) at the indicated concentrations. Cell viability was measured using Cell Titer-Glo luminescent cell viability assay. Data represent mean ± SEM of three independent experiments (**p < 0.01). [file 1479-5876-11-280-S7.jpeg]

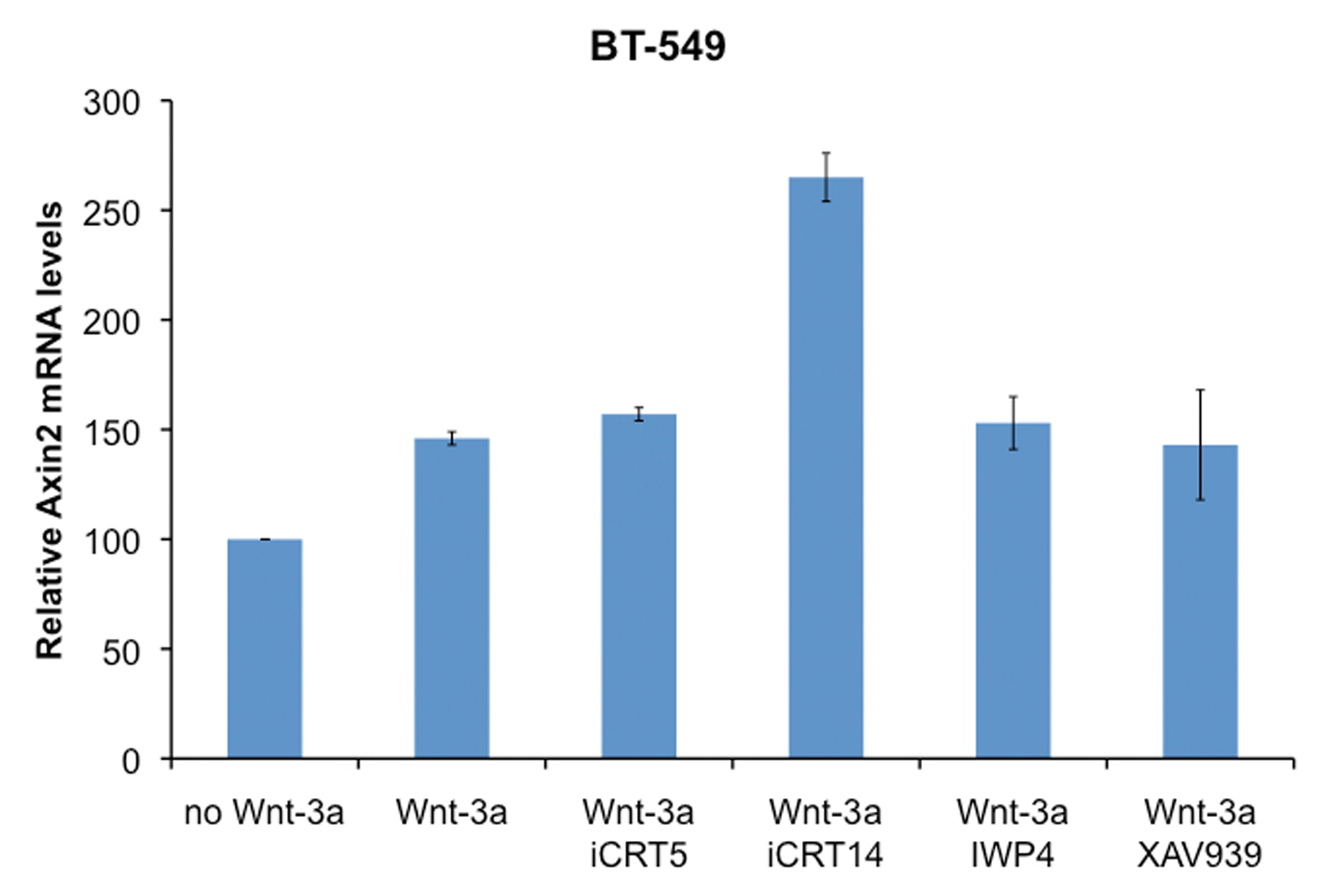

Supplement: Additional file 8: Figure S7 — Wnt pathway is not antagonized by iCRT-5, iCRT-14, IWP-4 or XAV-939. BT-549 cells were serum-starved for 24 hours, and treated with Wnt-3a (200 ng/ml) and/or iCRT-5 (50 μM), iCRT-14 (10 μM), IWP-4 (1 μM) or XAV-939 (5 μM) for 4 hours. Total RNA was prepared, and assessed for Axin2 expression using quantitative real-time RT-PCR. β-actin was used as normalization control. [file 1479-5876-11-280-S8.jpeg]
